# Latent ruthenium–indenylidene catalysts bearing a *N*-heterocyclic carbene and a bidentate picolinate ligand

**DOI:** 10.3762/bjoc.11.169

**Published:** 2015-09-03

**Authors:** Thibault E Schmid, Florian Modicom, Adrien Dumas, Etienne Borré, Loic Toupet, Olivier Baslé, Marc Mauduit

**Affiliations:** 1 Ecole Nationale supérieure de chimie de rennes, UMR CNRS 6226, 11 Allée de Beaulieu, 35708, Rennes, cedex 7, France; 2 Institut de Physique de Rennes, Université de Rennes 1, CNRS, UMR 6251, Rennes Cedex, France

**Keywords:** latent catalyst, olefin metathesis, picolinate ligand, ruthenium indenylidene

## Abstract

A silver-free methodology was developed for the synthesis of unprecedented *N*-heterocyclic carbene ruthenium indenylidene complexes bearing a bidentate picolinate ligand. The highly stable (SIPr)(picolinate)RuCl(indenylidene) complex **4a** (SIPr = 1,3-bis(2-6-diisopropylphenyl)imidazolidin-2-ylidene) demonstrated excellent latent behaviour in ring closing metathesis (RCM) reaction and could be activated in the presence of a Brønsted acid. The versatility of the catalyst **4a** was subsequently demonstrated in RCM, cross-metathesis (CM) and enyne metathesis reactions.

## Introduction

Olefin metathesis has witnessed tremendous development in the last decades and has emerged as a powerful tool with dramatic impact on both organic chemistry and materials science [1–2]. Intensive research has notably allowed for the design of efficient ruthenium-based catalysts that exhibit improved reactivity [3–6]. On the other hand, some applications require a precatalyst that can remain inert towards substrates and only initiate in response to a specific stimulus [7]. A common strategy for the preparation of latent catalysts is to incorporate additional strongly binding chelating ligands to the ruthenium coordination sphere [8]. Thus, activation of the catalyst is made possible by liberation of coordination vacancy under specific conditions, such as elevated temperature or addition of cocatalyst. While first examples of latent catalysts were based on phosphine-containing ruthenium complexes bearing a Schiff base ligand (O–N) [9] replacement of the phosphine ligand by sterically demanding and strongly σ-donor *N*-heterocyclic carbenes (NHC) afforded catalysts with improved catalytic performance [10–12]. Among the different [N–O]-chelating ligands that have been previously considered [13–17], the pyridyl-2-carboxylate (picolinate) ligand has demonstrated its usefulness in the preparation of efficient latent catalysts based on (NHC)Ru–alkylidene complexes [18–19]. Nevertheless, indenylidene complexes, that have notably showed an improved stability in comparison to their benzylidene counterparts [20–22], have never been considered in association with picolinic ligands for the synthesis of robust latent catalysts. Moreover, this strategy would provide an interesting alternative to indenylidene-Schiff base complexes that in most cases require toxic thallium salts for their preparation [23–24].

Here, we report the synthesis of (NHC)Ru–indenylidene complexes incorporating chelating picolinic ligands and evaluate their potential as latent catalysts in model ring closing metathesis and cross-metathesis transformations.

## Results and Discussion

**Scheme 1:**
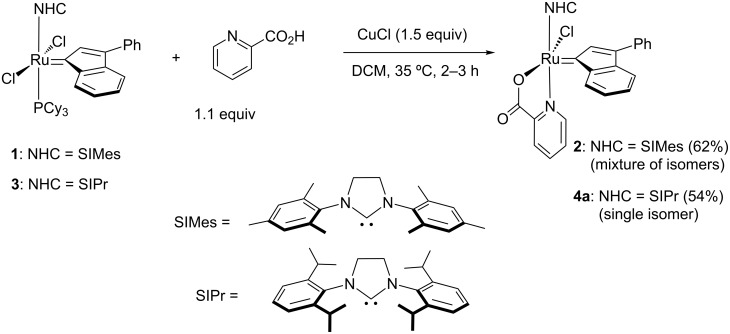
Synthesis of [NHC(picolinate)RuCl(indenylidene)] complexes **2** and **4a**.

With the objective to develop an attractive strategy for the synthesis of indenylidene-picolinic ruthenium complexes, we initially attempted their preparation using a silver-free methodology. In fact, silver residues are known to induce ruthenium-complex decomposition, and increase purification complexity [25]. Moreover, in a previous report regarding the synthesis of a dormant ruthenium catalyst bearing a chelating carboxylate ligand, spontaneous chloride/carboxylate exchange with elimination of HCl has been established [26]. Therefore, using only CuCl as a phosphine scavenger, we investigated the picolinic acid addition using the commercially available M2 complex **1** [27], featuring the NHC SIMes (SIMes = 1,3-bis(2,4,6-trimethylphenyl)-4,5-dihydroimidazol-2-ylidene) as a precursor. To our delight, using only 1.1 equiv of 2-picolinic acid, complex **2**, resulting from the single Cl/picolinate exchange, was identified as the major product (Scheme 1). Column chromatography enabled the purification of compound **2**, which could be isolated as a mixture of isomers with 62% yield. Encouraged by these preliminary results, we considered the preparation of a SIPr-based species, which would generate complexes with a higher catalytic potential and an improved stability [6,28–29]. Gratifyingly, under the same reaction conditions, the desired complex **4a** could be isolated as a single isomer in 54% yield.

**Scheme 2:**
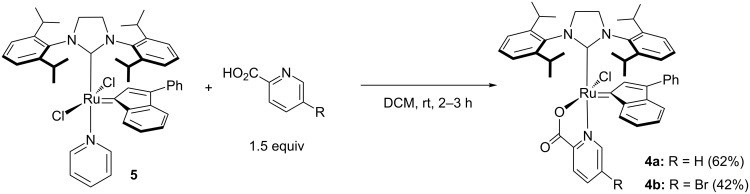
Synthesis of complex **4a** and **4b** from (SIPr)(pyridine)RuCl_2_(indenylidene) (**5**).

**Figure 1:**
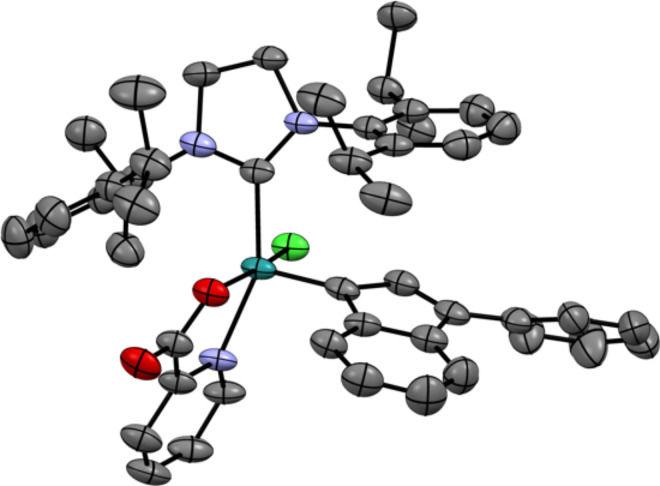
Solid-state structure of complex **4a** from single crystal X-ray diffraction. Hydrogens have been omitted for clarity. (N in blue, C in grey, O in red, Cl in green).

In order to improve the synthesis efficiency of the desired complex **4a**, the [(SIPr)RuCl_2_(indenylidene)pyridine] complex **5** was used as a precursor (Scheme 2). The latter species offers both the advantages to avoid the use of phosphine scavenger and to trap the acid formed as a pyridinium chloride salt. Gratifyingly, under these modified conditions the desired complex **4a** could be prepared in a clean manner with an improved 62% isolated yield. Given the importance of the substitution pattern of the chelating [N–O] ligand, X-ray diffraction analysis unambiguously confirmed the structure of complex **4a**, which displayed a distorted square pyramidal geometry (Figure 1) [30]. The nitrogen atom of the picolinate occupied a position *trans* to the bulky NHC ligand, with the carboxylate group *cis* to the indenylidene. In order to determine the influence of the electronic parameters of the chelating pseudo-halide fragment, we proceeded to synthesize complex **4b**, featuring the 5-bromo-2-picolinate moiety. Using a similar methodology, the desired complex was obtained with 42% yield.

**Figure 2:**
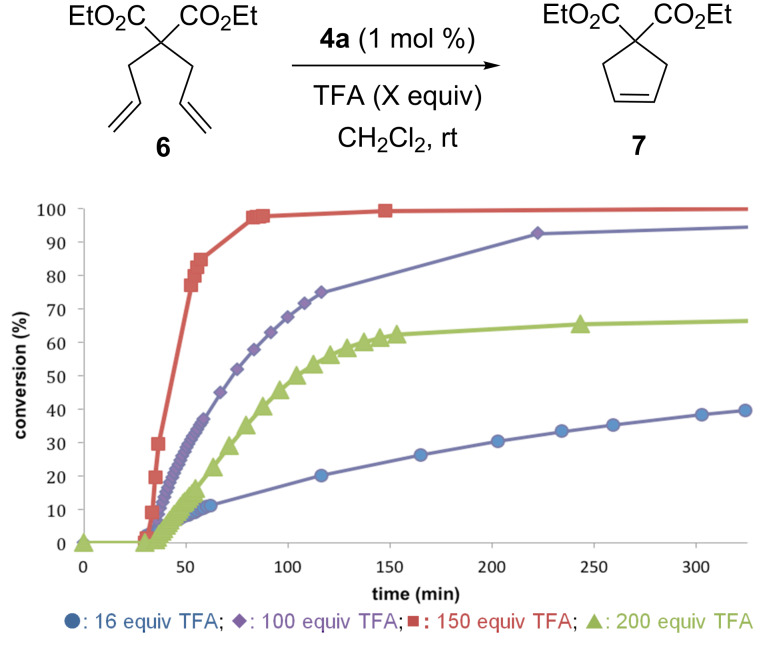
Olefin metathesis profiles in response to TFA equivalents.

The activation of the new picolinic complex **4a** was initially evaluated in the presence of trifluoroacetic acid (TFA), a Brønsted acid easy to handle and to operate (Figure 2). Consistent with its stability in solution, the latent catalyst **4a** appeared totally inactive at room temperature (<1% conversion after 24 h at 297 K), while catalytic activity was observed in the RCM of diethyl diallylmalonate (DEDAM) after addition of TFA. Several acid/catalyst ratios were investigated, and 150 equiv of TFA afforded the best catalytic performance in terms of initiation rate and conversion. In fact, both a decrease (100 equiv) and an increase (200 equiv) of this acid/catalyst ratio led to a significant deterioration of the kinetic profile.

**Figure 3:**
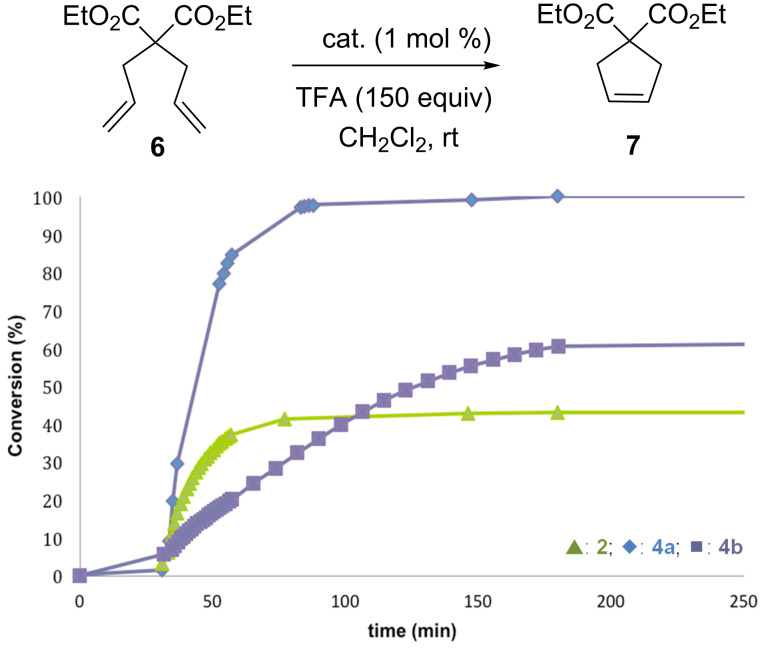
Comparison of olefin metathesis profiles for catalysts **2**, **4a** and **4b** after activation with 150 equiv of TFA.

Subsequently, the SIMes-based complex **2** and the 4-bromo-substituted complex **4b** were evaluated and compared with complex **4a** under these optimized reaction conditions (Figure 3). While in the absence of acid, **2** displayed no catalytic activity after 30 min, the complex **4b** bearing a more electron-deficient picolinate ligand demonstrated modest latency potential with 5% conversion after this period. In both cases, activation of the catalysts with 150 equiv of TFA did not provide full conversion of the benchmark substrate. Notably, despite a faster initiation rate, the SIMes-based catalyst **2** afforded a lower conversion rate, that can be explained by a rapid decomposition of the corresponding active species. Therefore, the complex **4a** that displayed the best catalytic performance was selected for further investigations.

**Figure 4:**
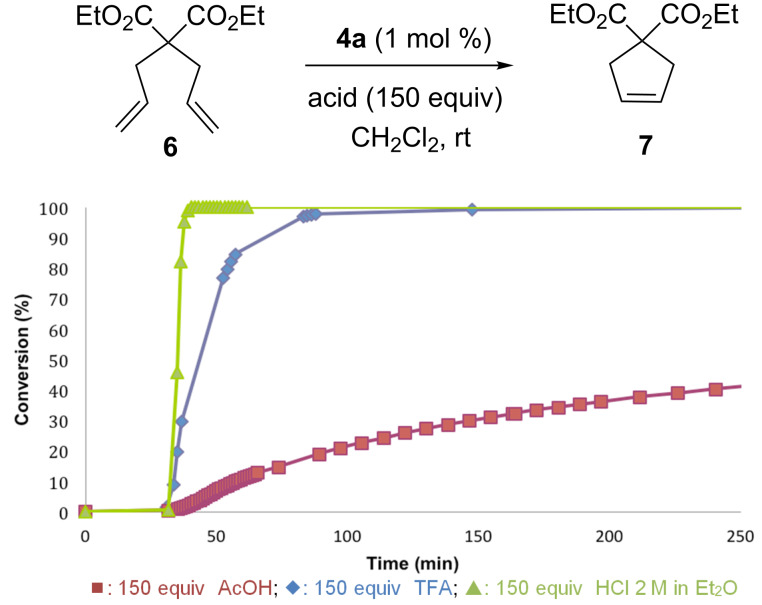
Influence of various acids for the activation of **4a** in the RCM of DEDAM.

Efforts were then focused to determine the effect of different acids on the catalyst efficiency in RCM of DEDAM [31]. The replacement of TFA by a weaker acid, acetic acid afforded a lower initiation rate and a conversion of only 40% was obtained after 5 h. On the other hand, hydrochloric acid outperformed TFA to complete the reaction in 10 min, as depicted in Figure 4.

With the best conditions in hand, we then briefly investigated the scope of metathesis transformations using 1 mol % of **4a** in dichloromethane at 25 °C, using hydrochloric acid as activator (Table 1). Both substrates **6** and the tosylamine **8** were efficiently converted to the desired cyclopentene products that were isolated in 97 and 96% yields, respectively. Interestingly, the more sterically-demanding diene **10** afforded the trisubstituted olefin cyclized product with high 93% isolated yield. Catalyst **4a** was also efficient regarding the cyclization of enynes **12** and **14** and the desired diene products were obtained with 89 and 90% isolated yields, respectively. Finally, the cross metathesis reaction between allylbenzene and ethyl acrylate afforded the desired internal *E*-olefin with 62% yield.

**Table 1:** Substrate scope in RCM, CM and enyne metathesis catalyzed by **4a**^a^.

Entry	Substrate	Product	Yield (%)

1	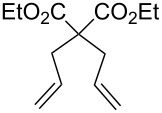 **6**	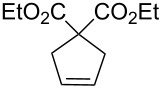 **7**	97
2	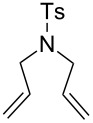 **8**	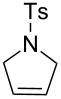 **9**	96
3	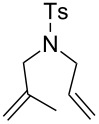 **10**	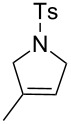 **11**	93
4	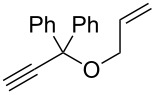 **12**	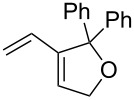 **13**	89
5	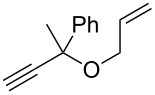 **14**	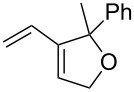 **15**	90
6	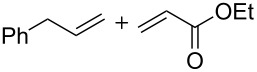 **16** + **17**	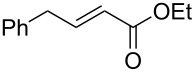 **18**	62

^a^Reaction conditions: substrate (0.238 mmol), CH_2_Cl_2_ (2.4 mL), **4a** (0.00238 mmol, injected from stock solution in CH_2_Cl_2_), HCl (0.357 mmol, injected from a 2 M solution in Et_2_O), room temperature, 2 h.

## Conclusion

We described the synthesis and characterization of three novel latent 2^nd^ generation indenylidene-based precatalysts for olefin metathesis reactions. A picolinate moiety was successfully inserted into SIMes- and SIPr-containing architectures, affording unprecedented mono-picolinate complexes. Whilst SIMes-containing species **2** was identified as a mixture of isomers with a low stability in solution, compounds **4a** and **4b**, featuring the bulkier ancillary ligand SIPr, appeared as highly stable, well-defined species. These species were shown to activate in the presence of a Brønsted acid and precatalysts **2** and **4a** showed an excellent latent behaviour. The best reaction rates were obtained in the RCM of DEDAM with complex **4a** after activation by HCl addition. The versatility of the catalyst was subsequently demonstrated in RCM, CM and enyne metathesis reactions.

## Supporting Information

File 1 Full experimental procedures and detailed analytical data for ruthenium complexes.
