# Supplementary material for: Latent ruthenium–indenylidene catalysts bearing a N-heterocyclic carbene and a bidentate picolinate ligand
Source: Beilstein J Org Chem. 2015 Sep 3;11:1541–6. doi: 10.3762/bjoc.11.169 (PMC4578404; doi:10.3762/bjoc.11.169)
Supplement: File 1 — Full experimental procedures and detailed analytical data for ruthenium complexes. [file Beilstein_J_Org_Chem-11-1541-s001.pdf]

Supporting Information
for

Latent ruthenium–indenylidene catalysts bearing a *N*-heterocyclic carbene and a bidentate picolinate ligand.

Thibault, E. Schmid[1], Florian Modicom[1], Adrien Dumas[1], Etienne Borré[1], Loic Toupet[2], Olivier Baslé*[1] and Marc Mauduit*[1]

Address: [1]Ecole Nationale supérieure de chimie de rennes, UMR CNRS 6226, 11 Allée de Beaulieu, 35708, Rennes, cedex 7, France and [2]Institut de Physique de Rennes, Université de Rennes 1, CNRS, UMR 6251, Rennes Cedex, France

Email: Olivier Baslé - olivier.basle@ensc-rennes.fr; Marc Mauduit - marc.mauduit@enscr-rennes.fr

*Corresponding author

Full experimental procedures and detailed analytical data for ruthenium complexes.

General s2

Synthesis and analytical data of chloro-(2-pyridinecarboxylato)(1,3-dimesitylimidazolidin-2-ylidene))(3-phenyl-1*H*-inden-1-ylidene) ruthenium(II) (**2**). s2

Synthesis and analytical data of chloro-(2-pyridinecarboxylato)(1,3-bis(2,6-diisopropylphenyl)imidazolidin-2-ylidene)) (3-phenyl-1*H*-inden-1-ylidene) ruthenium(II) (**4a**). s3

Synthesis and analytical data of chloro-(5-bromo-2-pyridinecarboxylato) - (1,3-bis(2,6-diisopropylphenyl)imidazolidin-2-ylidene))(3-phenyl-1*H*-inden-1-ylidene) ruthenium(II) (**4b**). s4

NMR Spectra of new complexes s6

Determination of the acid quantity influence in the RCM of DEDAM with **4a** . s9

Catalyst comparaison in the RCM of DEDAM with TFA s9

Acid comparison in the RCM of DEDAM with **4a** s9

General procedure for metathesis reactions s10

X-ray data for **4a** s11

General

All the reactions were carried out under inert atmosphere (argon). Dichloromethane was distilled over calcium hydride prior to use. Tetrahydrofuran was distilled over sodium/benzophenone prior to use. All commercial chemicals were used as received unless otherwise noted. ^{1}H (400 MHz) and ^{13}C (100 MHz) NMR spectra were recorded on a Bruker ARX400 spectrometer with complete proton decoupling for ^{13}C. ^{1}H and ^{13}C chemical shifts are reported in parts per million with the solvent resonance as the internal standard ($CDCl_3$, ^{1}H: δ 7.26 ppm, ^{13}C: δ 77.16 ppm; CD_2Cl_2, ^{1}H: δ 5.31, ^{13}C: δ 53.8 ppm). Coupling constants are reported in Hertz (Hz). Abbreviations are used as follows: s = singlet, d = doublet, t = triplet, q = quadruplet, sep = septet, m = multiplet, br = broad. High-resolution mass spectrometry (HMRS) analyses were performed at the Centre Régional de Mesures Physiques de l'Ouest (CRMPO), Université de Rennes 1.

Synthesis and analytical data of chloro-(2-pyridinecarboxylato)(1,3-dimesitylimidazolidin-2-ylidene))(3-phenyl-1*H*-inden-1-ylidene) ruthenium(II) (2).

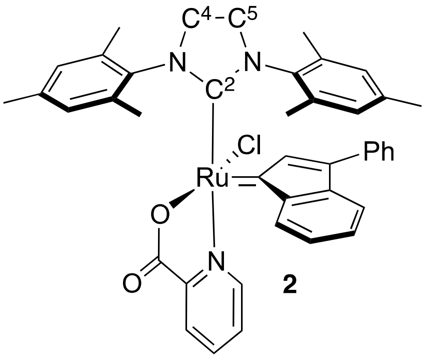

2

In a flame-dried Schlenk tube equipped with a stirring bar, complex **1** (500.0 mg, 0.53 mmol), 2-picolinic acid (71.4 mg, 0.58 mmol) and copper(I) chloride (97%, 80.7 mg, 0.79 mmol) were added. The Schlenk tube was then submitted to 3 argon–vacuum cycles after which 12 mL of CH_2Cl_2 were added. The reaction mixture was stirred for 2 h at 35 °C after what it was evaporated to dryness. The solid was dissolved in acetone and filtrated on a short plug of celite to remove the copper salts. The filtrate was concentrated under reduced pressure and the crude material was then purified by flash chromatography on silica gel (pentane/acetone, 9:1 to 8:2), affording compound **2** as a red powder in 62% yield (272.0 mg, 0.36 mmol).

^{1}H NMR (400 MHz, $CDCl_3$): δ (ppm) 1.83 (br s, 3H, Ar-CH_3), 1.98 (br s, 3H, Ar-CH_3), 2.06-2.16 (m, 3H, Ar-CH_3), 2.23-2.34 (m, 3H, Ar-CH_3), 2.51 (br s, 3H, Ar-CH_3), 2.65 (br s, 3H, Ar-CH_3), 4.12-4.59 (m, 4H, C^4-H, C^5-H), 6.04-6.13 (m, 1H, Ar-H), 6.43-6.53 (m, 1H, Ar-H), 6.71-6.83 (m, 2H, Ar-H), 6.95-6.99 (m, 1H, Ar-H), 7.17-7.18 (m, 2H, Ar-H), 7.29-7.39 (m, 3H, Ar-H), 7.48-7.50 (m, 2H, Ar-H), 7.65 (d, $^3J_{H-H}$ = 7.2 Hz, 2H, Ar-H), 7.78-7.80 (m, 1H, Ar-H), 7.90 (br s, 1H, Ar-H), 8.21-8.52 (m, 1H, Ar-H), 8.69-8.83 (m, 1H, Ar-H).

Due to the instability of compound **2** in solution, a ^{13}C NMR spectra of sufficient quality could not be recorded.

HRMS (ESI) : m/z : $[M]^+$ ($C_{42}H_{40}N_3O_2{}^{35}Cl^{102}Ru$) calc. 755.1847 ; found 755.1833 (2 ppm)

Synthesis and analytical data of chloro-(2-pyridincarboxylato)(1,3-bis(2,6-diisopropylphenyl)imidazolidin-2-ylidene))(3-phenyl-1*H*-inden-1-ylidene) ruthenium(II) (4a).

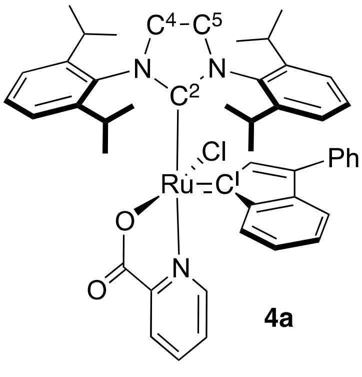

4a

In a flame-dried Schlenk tube equipped with a stirring bar, complex **5** (100.0 mg, 0.12 mmol) and 2-picolinic acid (22.2 mg, 0.18 mmol) were added. The Schlenk tube was then submitted to 3 argon–vacuum cycles after which 2 mL of CH_2Cl_2 were added. The reaction mixture was stirred for 3 hours after which it was evaporated to dryness. The crude mixture was then purified by flash chromatography on silica gel (pentane/acetone, 8/2), affording compound **4a** as a red powder in 62% yield (62 mg, 0.073 mmol). Crystals suitable for X-ray diffraction were obtained by slow diffusion of pentane in a solution of **4a** in CH_2Cl_2 (*ca*. 5 mg in 0.5 mL CH_2Cl_2, 2 mL pentane).

For the NMR analysis, 8.4 mg (0.01 mmol) of the complex was dissolved in 0.6 mL CD_2Cl_2. A stock solution of mesitylene (40.0 mg) in CD_2Cl_2 (1 mL) was prepared and 10 μL (0.0033 mmol) of this solution was added to the NMR tube through a septum under argon. This procedure resulted in an

improvement of the resolution, the ^{1}H NMR is therefore reported in the presence of mesitylene.

^{1}H NMR (400 MHz, CD_2Cl_2 in the presence of mesitylene) : δ (ppm) 1.08 (br d, $^{3}J_{H\text{-}H}$ = 5.6 Hz, 6H, CH-C*H*$_3$), 1.15 (d, $^{3}J_{H\text{-}H}$ = 7.2 Hz, 6H, CH-C*H*$_3$), 1.18 (d, $^{3}J_{H\text{-}H}$ = 6.8 Hz, 6H, CH-C*H*$_3$), 1.37 (br d, $^{3}J_{H\text{-}H}$ = 3.6 Hz, 6H, CH-C*H*$_3$), 3.33-3.73 (m, 4H, C*H*-CH_3), 4.23-4.37 (m, 4H, C^{4}-H, C^{5}-H), 6.20 (s, 1H, Ind-H), 6.84 (d, $^{3}J_{H\text{-}H}$ = 7.6 Hz, 2H, Ar-H), 6.95-7.09 (m, 6H, Ar-H), 7.16 (td, $^{3}J_{H\text{-}H}$ = 7.4 Hz, $^{4}J_{H\text{-}H}$ = 1.2 Hz, 1H, Ar-H), 7.31-7.35 (m, 3H, Ar-H), 7.47-7.52 (m, 1H, Ar-H), 7.53-7.55 (m, 2H, Ar-H), 7.69 (d, $^{3}J_{H\text{-}H}$ = 7.2 Hz, 1H, Ar-H), 7.75 (td, $^{3}J_{H\text{-}H}$ = 7.6, $^{4}J_{H\text{-}H}$ = 1.6 Hz, 1H, Ar-H), 8.11 (br d, $^{3}J_{H\text{-}H}$ = 7.2 Hz, 1H, Ar-H), 8.72 (br s, 1H, Ar-H).

^{13}C NMR (100 MHz, CD_2Cl_2) : δ (ppm) 22.7 (s), 22.9 (s), 27.1 (s), 29.0 (s), 29.1 (s), 54.6 (s), 117.6 (s), 123.9, (s) 124.7, (s), 125.1 (s), 126.3 (s), 126.9 (s), 127.3 (s), 128.2 (s), 129.0 (s), 129.7 (s), 130.2 (s), 134.7 (br s), 136.5 (s), 138.0 (br s), 139.5 (br s), 140.3 (s), 141.5 (s), 143.1 (s), 148.3 (br s), 148.8 (s), 149.4 (br s), 151.1 (s), 172.8 (s), 217.2 (s, C^{2}), 298.6 (s, C^{I}).

HRMS (ESI) : m/z : $[M+H]^{+}$ ($C_{48}H_{53}N_3O_2{}^{35}Cl^{102}Ru$) calc. : 840.28698 ; found : 840.2880 (1 ppm).

Synthesis and analytical data of chloro-(5-bromo-2-pyridincarboxylato)(1,3-bis(2,6-diisopropylphenyl)imidazolidin-2-ylidene))(3-phenyl-1*H*-inden-1-ylidene) ruthenium (II) 4b.

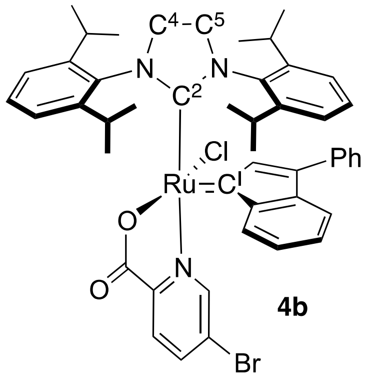

4b

In a flame-dried Schlenk tube equipped with a stirring bar, complex **5** (100.0 mg, 0.12 mmol) and 5-bromopyridine-2-carboxylic acid (36.4 mg, 0.18 mmol) were added. The Schlenk tube was then submitted to 3 argon–vacuum cycles after what 2 mL of CH_2Cl_2 were added. The reaction was stirred for 2 hours after what it was evaporated to dryness. The crude mixture was then purified by flash chromatography on silica gel (pentane/acetone, 8/2), affording compound **4b** as a brownish powder in 43% yield (47 mg, 0.051 mmol).

For the NMR analysis, 9.6 mg (0.01 mmol) of the complex was dissolved in 0.6 mL CD_2Cl_2. A stock solution of mesitylene (40.4 mg) in CD_2Cl_2 (1 mL) was prepared and 10 μL (0.0033 mmol) of this solution was added to the NMR tube through a septum under argon. This procedure resulted in an improvement of the resolution, the ^{1}H NMR is therefore reported in the presence of mesitylene.

^{1}H NMR (400 MHz, CD_2Cl_2 in the presence of mesitylene) : δ (ppm) 1.08 (br d, $^3J_{H\text{-}H}$ = 5.6 Hz, 6H, CH-C*H₃*), 1.15 (d, 6H, $^3J_{H\text{-}H}$ = 6.8 Hz, CH-CH_3), 1.18 (d, $^3J_{H\text{-}H}$ = 6.8 Hz, 6H, CH-CH_3), 1.37 (br d, $^3J_{H\text{-}H}$ = 5.2 Hz, 6H, CH-CH_3), 3.33-3.70 (m, 4H, C*H*-CH_3), 4.24-4.35 (m, 4H, C^4-H, C^5-H), 6.15 (s, 1H, Ind-H), 6.60 (d, $^3J_{H\text{-}H}$ = 7.2 Hz, 2H, Ar-H), 6.95-7.03 (m, 5H, Ar-H), 7.09 (td, $^3J_{H\text{-}H}$ = 7.2 Hz, $^4J_{H\text{-}H}$ = 0.8 Hz, 1H, Ar-H), 7.17 (td, $^3J_{H\text{-}H}$ = 7.4 Hz, , $^4J_{H\text{-}H}$ = 0.8 Hz, 1H, Ar-H), 7.31-7.35 (m, 2H, Ar-H), 7.48-7.59 (m, 4H, Ar-H), 7.87 (dd, $^3J_{H\text{-}H}$ = 8.4 Hz, $^4J_{H\text{-}H}$ = 2.0 Hz, 1H, Ar-H), 8.05 (br d, $^3J_{H\text{-}H}$ = 7.2 Hz, 1H, Ar-H), 8.77 (br s, 1H, Ar-H).

^{13}C NMR (100 MHz, CD_2Cl_2) : δ (ppm) 22.6 (s), 22.8 (s), 27.1 (s), 29.0 (s), 29.1 (s), 54.5 (s), 117.7 (s), 123.1 (s), 124.0 (s), 124.7 (s), 126.2 (s), 126.9 (s), 127.3 (s), 128.3 (s), 129.0 (s), 129.2 (s), 129.9 (s), 130.3 (s), 134.5 (br s), 136.3 (s), 138.3 (s), 141.4 (s), 142.9 (s), 143.1 (s), 148.3 (s), 149.5 (s), 149.8 (s), 150.0 (s), 172.1 (s), 216.1 (s, C^2), 300.1 (s, C^I).

HRMS (ESI) : m/z : $[M]^+$ ($C_{48}H_{51}N_3O_2{}^{35}Cl^{79}Br^{102}Ru$) calc. 917.18966 ; found 917.1915 (2 ppm) ; $[M\text{-}Cl]^+$ ($C_{48}H_{51}N_3O_2{}^{79}Br^{102}Ru$) calc. 882.22081 ; found 882.2254 (5 ppm).

NMR Spectra of new complexes

^{1}H NMR of complex **2** ($CDCl_3$, 400 MHz)

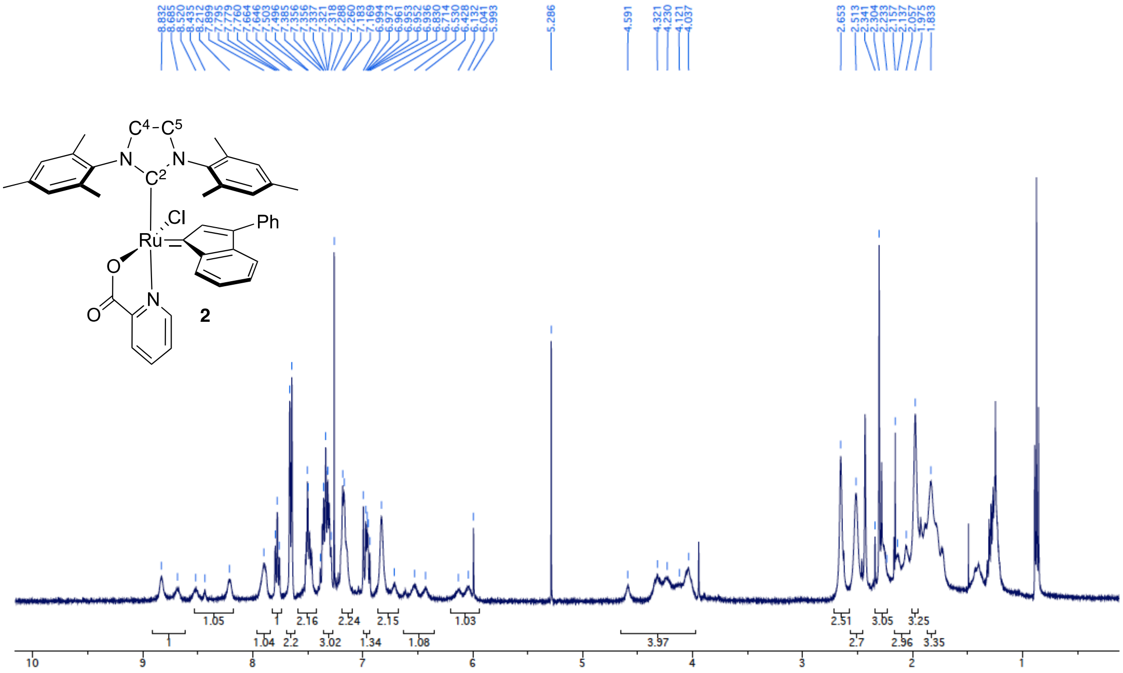

^{1}H NMR of complex **4a** in the presence of mesitylene (CD_2Cl_2, 400 MHz)

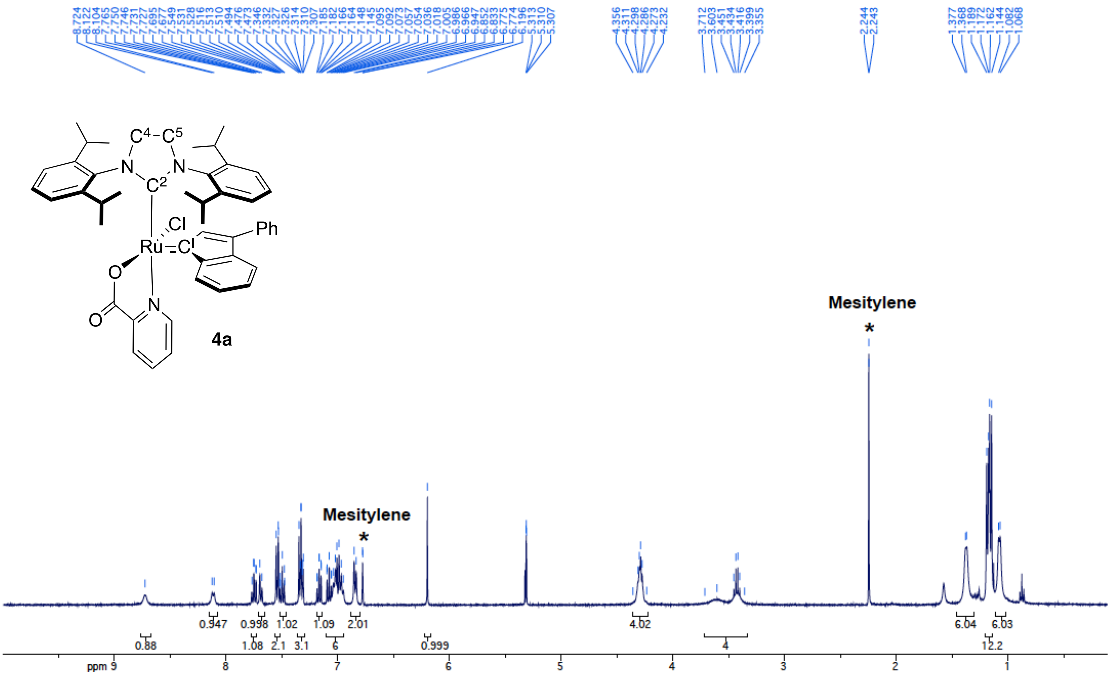

^{13}C NMR of complex **4a** (CD_2Cl_2, 400 MHz)

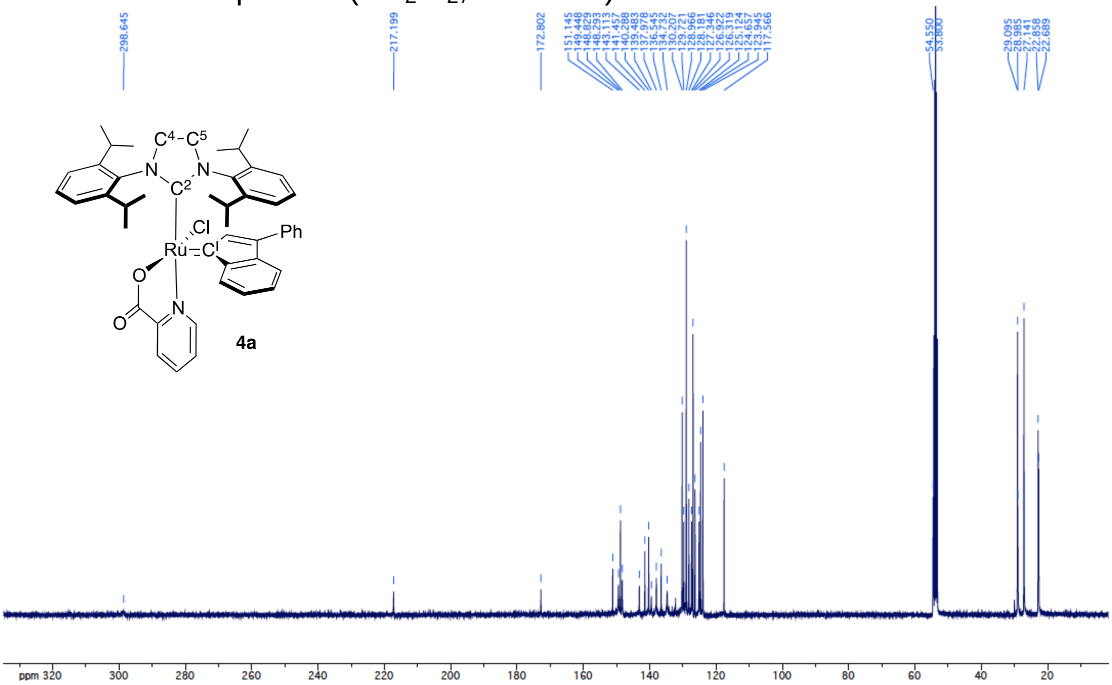

^{1}H NMR of complex **4b** in the presence of mesitylene (CD_2Cl_2, 400 MHz)

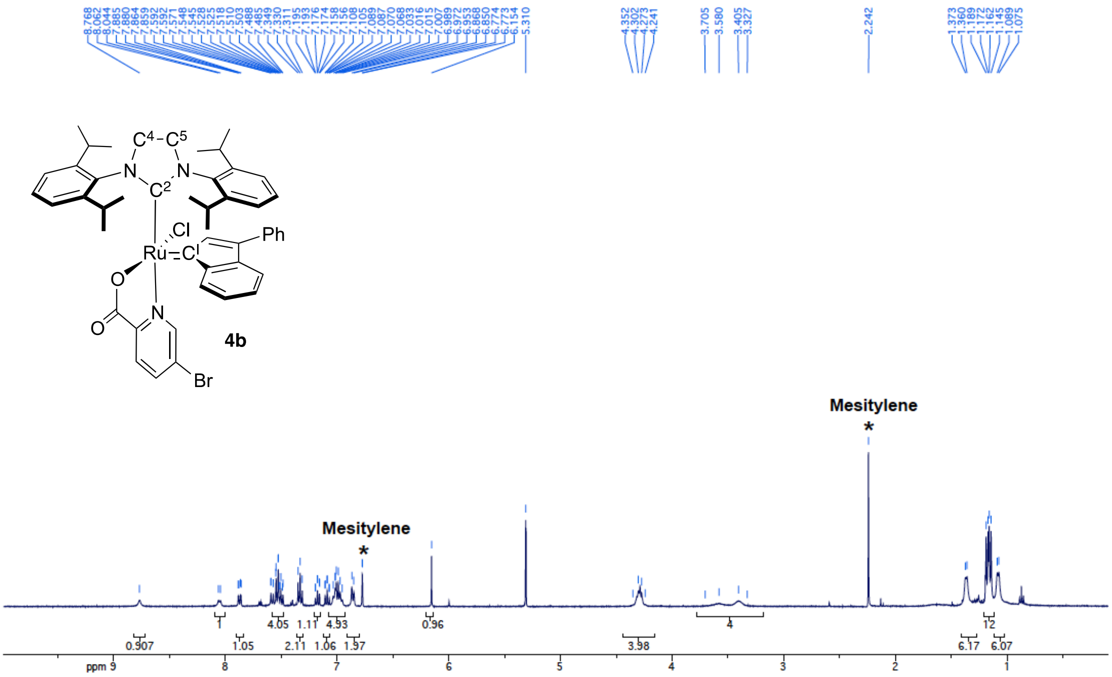

^{13}C NMR of complex **4b** (CD_2Cl_2, 400 MHz)

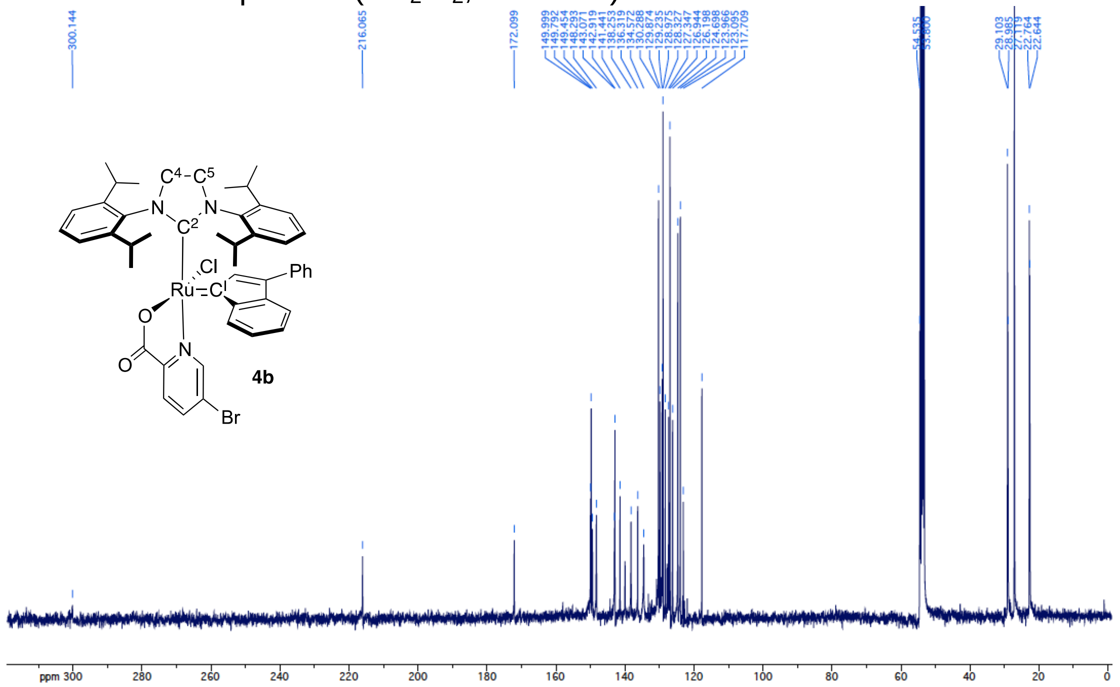

Determination of the acid quantity influence in the RCM of DEDAM with 4a

In a flame-dried NMR tube under Argon, DEDAM (19 μL, 0.08 mmol), mesitylene (11 μL, 0.08 mmol) and CD_2Cl_2 (800 μL) were added. A solution of catalyst **4a** in CD_2Cl_2 was prepared in a separate flask under Argon and the catalyst was injected in the NMR tube (0.0008 mmol). After 30 minutes, an NMR spectrum was recorded after what the tube was ejected and TFA (between 1 and 12 μL) was added. NMR spectra were then taken at regular intervals and the reaction conversion was determined with the integral ratio of the reaction product with mesitylene.

Catalyst comparison in the RCM of DEDAM with TFA

In a flame-dried NMR tube under Argon, DEDAM (19 μL, 0.08 mmol), mesitylene (11 μL, 0.08 mmol) and CD_2Cl_2 (800 μL) were added. A solution of catalyst in CD_2Cl_2 was prepared in a separate flask under Argon and the catalyst was injected in the NMR tube (0.0008 mmol). After 30 minutes, an NMR spectrum was recorded after what the tube was ejected and TFA (9 μL, 0.12 mmol) was added. NMR spectra were then taken at regular intervals and the reaction conversion was determined with the integral ratio of the reaction product with mesitylene.

Acid comparison in the RCM of DEDAM with 4a

In a flame-dried NMR tube under Argon, DEDAM (19 μL, 0.08 mmol), mesitylene (11 μL, 0.08 mmol) and CD_2Cl_2 (800 μL) were added. A solution of catalyst **4a** in CD_2Cl_2 was prepared in a separate flask under Argon and the catalyst was injected in the NMR tube (0.0008 mmol). After 30 minutes, an NMR spectrum was recorded after what the tube was ejected and the acid (0.12 mmol) was added. NMR spectra were then taken at regular intervals and the reaction conversion was determined with the integral ratio of the reaction product with mesitylene.

General procedure for metathesis reactions

In a flame-dried round bottomed flask, catalyst **4a** (2 mg, 0.00238 mmol) was added and the flask was submitted to 3 cycles of vaccum–argon. CH_2Cl_2 (2,4 mL) was added and the substrate (0.238 mmol) was injected. A 2 M solution of HCl in Et_2O (180 μL, 0.357 mmol) was added and the mixture was stirred at room temperature for 2 h. The conversion was controlled by TLC (Pentane/Et_2O 95/5). After complete conversion, the solvent was removed under vaccum and the crude mixture was purified on silica gel (pentane/Et_2O, 95:5), affording the pure product. The ^1H NMR spectra of the obtained products for entries 1,[1] 2,[2] 3,[3] 4,[2] 5,[4] and 6[5] were found to be in accordance with the literature.

---

[1] W. A. Nugent, J. Feldman, J. C. Calabrese, *J. Am. Chem. Soc*., **1995**, *117*, 8992-8998.
[2] A. Michrowska, K. Mennecke, U. Kunz, A. Kirsching, K. Grela, *J. Am. Chem. Soc.*, **2006**, *128*, 13261–13267
[3] F. Michalek, D. Mädge, J. Mühe, W. Bannwarth, *J. Organomet. Chem.*, **2006**, *691*, 5172-5180
[4] T. E. Schmid, X. Bantreil, C. Citadelle, A. M. Z. Slawin, C. S. J. Cazin, *Chem. Commun.*, **2011**, *47*, 7060-7062.
[5] A. A. Poeylaut-Palena, S. A. Testero, E. G. Mata, *J. Org. Chem.*, **2008**, *73*, 2024-2027

X-ray data for 4a

CCDC 1405734

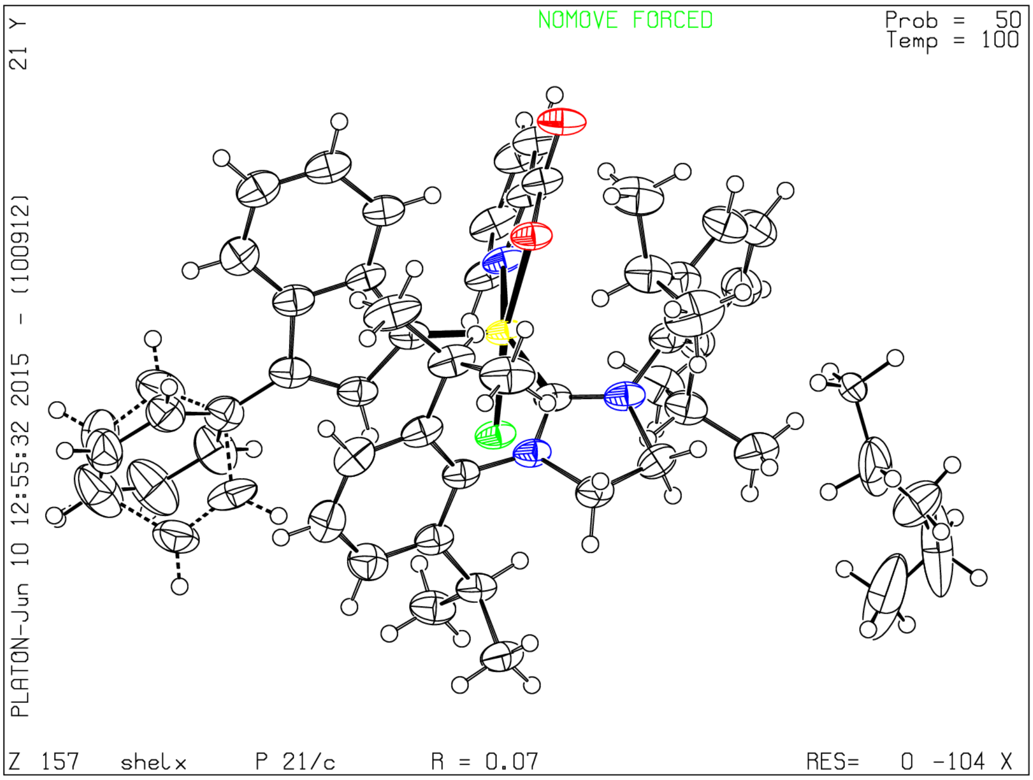

Identification code	shelx	
Empirical formula	C50.50 H58 Cl N3 O2 Ru	
Formula weight	875.52	
Temperature	100(2) K	
Wavelength	1.54184 A	
Crystal system, space group	Monoclinic, P 21/c	
Unit cell dimensions	a = 13.3934(6) A	alpha = 90 deg.
	b = 20.0867(4) A	beta = 99.927(3) deg.
	c = 17.5572(4) A	gamma = 90 deg.
Volume	4652.7(3) A^3	
Z, Calculated density	4, 1.250 Mg/m^3	
Absorption coefficient	3.563 mm^-1	
F(000)	1836	

Crystal size	0.172 x 0.130 x 0.054 mm
Theta range for data collection	3.350 to 70.951 deg.
Limiting indices	-16<=h<=16, -24<=k<=24, -20<=l<=21
Reflections collected / unique	61346 / 8924 [R(int) = 0.0909]
Completeness to theta = 67.684	100.0 %
Absorption correction	Semi-empirical from equivalents
Max. and min. transmission	1.00000 and 0.71311
Refinement method	Full-matrix least-squares on F^2
Data / restraints / parameters	8924 / 4 / 579
Goodness-of-fit on F^2	1.015
Final R indices [I>2sigma(I)]	R1 = 0.0665, wR2 = 0.1812
R indices (all data)	R1 = 0.0823, wR2 = 0.1929
Extinction coefficient	0.00011(4)
Largest diff. peak and hole	2.784 and -0.870 e.A^-3
